# 

**Published:** 1894-02

**Authors:** 


### The American Medical Association.

This body meets at San Francisco on June 5, 1894. This occurs during the Mid-Winter Fair. On account of this and many other attractions doubtless there will be a large attendance of medical men, and we desire especially that there shall be a large representation of dentists from this side of the Rocky Mountains, who will go and insure a large dental section. We are assured that the dentists of California are making large preparation for this occasion; they are doing all they can to make it pleasant, interesting and profitable to their brethren of the East who may be present.

An opportunity will be afforded for observation and sight-seeing that likely will never be realized again. San Francisco and other places and objects of interest will be available for sight-seers.

Railroad rates for the round trip will be very low, so that many inducements will be presented for the medical profession in all its departments to visit California at that time. We make this timely suggestion in order that the largest number may take time by the forelock, and be prepared to enjoy the journey and the occasion.

We shall again give more definite information when further details come to hand.

### THE DENTAL REGISTER,

**A MONTHLY MAGAZINE DEVOTED TO THE INTERESTS OF THE**

**DENTAL PROFESSION.**

*Terms Reduced to* $2.00 *Per Year. Single Copies,* 25 *Cents.*

**EDITED BY PROF. J. TAFT, D.D.S.**

—AND—

**Published by SAM'L A. CROCKER & CO., Ohio Dental and Surgical Depot.**

The volume commences in January and closes in December.

The REGISTER being issued monthly is always fresh, therefore Indispensable to the practitioner who desires to keep posted up with the new inventions and improvements which are daily developed. Neither expense nor effort will be spared to give value and variety to its contents Articles will be illustrated with well executed engravings whenever they will add to the interest or assist to explanation.

Its extensive circulation and frequency of issue make it one of the BEST DENTAL ADVERTISING MEDIUMS in the United States.

All subscriptions must begin with January or July.

Local or special notices, five lines or less, will be inserted for $3 each insertion ; over five lines, 50 cts. per line.

All advertising bills payable in advance, or at furthest, after the issue of the first number containing the advertisement. All transient advertisements to be paid for in advance.

☞CONTRIBUTIONS SOLICITED.

Exclusively Editorial Communications should be addressed to the Editor.

The latest number of the REGISTER may be ordered with or without other good at any time, and all letters should be addressed to

